# The Impact of CRISPR-Cas System on Antiviral Therapy

**DOI:** 10.15171/apb.2018.067

**Published:** 2018-11-29

**Authors:** Hadi Bayat, Fatemeh Naderi, Amjad Hayat Khan, Arash Memarnejadian, Azam Rahimpour

**Affiliations:** ^1^Department of Tissue Engineering and Applied Cell Sciences, School of Advanced Technologies in Medicine, Shahid Beheshti University of Medical Sciences, Tehran, Iran.; ^2^Department of Molecular Genetics, Faculty of Biological Sciences, Tarbiat Modares University, Tehran, Iran.; ^3^Department of Molecular Genetics, Tehran Medical Sciences Branch, Islamic Azad University, Tehran, Iran.; ^4^Institute for Research in Molecular Medicine (INFORMM), Universiti Sains Malaysia, Penang, Malaysia.; ^5^Department of Hepatitis and AIDS, Pasteur Institute of Iran, Tehran, Iran.

**Keywords:** CRISPR-Cas, Antiviral therapy, Genome editing, Latent viruses, Delivery system

## Abstract

Clustered regularly interspaced short palindromic repeats (CRISPR)-associated protein nuclease (Cas) is identified as an adaptive immune system in archaea and bacteria. Type II of this system, CRISPR-Cas9, is the most versatile form that has enabled facile and efficient targeted genome editing. Viral infections have serious impacts on global health and conventional antiviral therapies have not yielded a successful solution hitherto. The CRISPR-Cas9 system represents a promising tool for eliminating viral infections. In this review, we highlight 1) the recent progress of CRISPR-Cas technology in decoding and diagnosis of viral outbreaks, 2) its applications to eliminate viral infections in both pre-integration and provirus stages, and 3) various delivery systems that are employed to introduce the platform into target cells.

## Introduction

**Figure 1:**
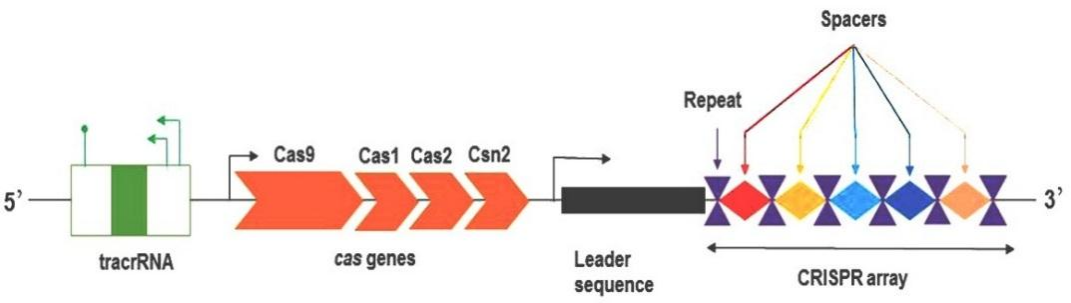
Schematic view of the type II-A CRISPR-Cas system. The components of the CRISPR system ordered from 5’ to 3’. tracrRNA: trans-activating crRNA.

The clustered regularly interspaced short palindromic repeats (CRISPR)-associated protein nuclease (Cas) is a prokaryotic antiviral adaptive immune system, which is present in most archaea (~90%) and some bacteria (~50%). The genomic components of the CRISPR system are made up of trans-activating crRNA (tracrRNA), the *cas* operon, a leader sequence and arrays of short direct repeats. These repeats are interspersed by non-repetitive spacer sequences, which are acquired from mobile invasive elements mainly viruses and plasmids (Figure 1). The CRISPR-Cas system confers the organism’s resistance against foreign genetic elements that have previously rendered parts of their genome spacer sequences into the CRISPR array. CRISPR-Cas9 system is derived from type II, the simplest and most commonly used system in genome editing approaches.^1^ Host codon-optimized Cas9 is recruited on target site by designable guide-RNA (gRNA) and precisely introduces double strand break (DSB) ~3-base pair (bp) upstream of the protospapcer adjacent motif (PAM). Then, the DSB is repaired with either the error-prone non-homologous end joining (NHEJ) or homology-directed repair (HDR) pathways. NHEJ leaves the genome vulnerable to a lethal genomic mutation, by frameshifting an open reading frame (ORF) on the target gene. Giant viruses also have a defense structure reminiscent of the CRISPR-Cas system. The viral defense system known as the mimivirus virophage resistance element (MIMIVIRE) is composed of proteins with both nuclease and helicase activities, representing an adaptive immune system based on nucleic acid against virophages.^2^ Over the recent years, CRISPR-Cas technologies have been well-optimized in eukaryotic cells, particularly in human cells.

Infectious viral diseases are serious global health concerns and despite the huge efforts invested in their eradication, only limited success has been achieved. Establishment of long-term infections leading to chronic disease and also development of antiviral resistant mutants are factors that lead to the persistent viral infections. Novel strategies are required to eliminate even traces of viruses within the host.^3^ During the last few years, the applications of CRISPR-Cas9 system have introduced novel antiviral therapeutic options. The advantage of CRISPR-Cas9 technology lies in their ability to directly target the viral DNA or RNA. In this line, the viral infection would be eliminated in the host. CRISPR-Cas systems have shown their efficacy in different viral infections in both pre-integration and provirus stages.^4^ Similarly, CRISPR-Cas has generated striking insights for development of novel vaccination strategies in poultry industry. It has been reported that CRISPR-Cas9 system can efficiently modify the genome of duck enteritis virus (DEV) C-KCE strain. The envelope glycoprotein gene and pre-membrane proteins of duck tembusu virus (DTMUV) as well as the hemagglutinin gene of highly pathogenic avian influenza virus (HPAIV) H5N1 were inserted at the suited sites in C-KCE strain to develop a trivalent vaccine that can efficiently prevent the infection of DTMUV, H5N1, and DEV in ducks.^5^ In addition to targeting DNA viruses, CRISPR-Cas9 system demonstrated its feasibility and versatility in targeting RNA viruses. Engineered *Francisella novicida* Cas9 (FnCas9) can successfully target positive-sense single strand RNA hepatitis C virus in eukaryotic cells. In contrast to *Streptococcus pyogenes* Cas9 (SpCas9) which needs synthesized PAM-encoding oligomer in targeting RNA *in vitro,* FnCas9 targets the RNA virus PAM-independently‏. In addition, the ability of FnCas9 to target RNA in cytosol can reduce off-target activity of the system on the host DNA compared to Cas9 which targets DNA in nuclear.^6^

In the current study, we recapitulated the CRISPR-Cas9 system impact on different kinds of viral genomes which can cause either detrimental acute or persistent infection in humans.

### Decoding and diagnosis of the obscure viruses

The rapid expansion of human flavivirus infections namely dengue virus (DENV) and Zika virus (ZIKV) have persuaded the research community to devise effective therapies against them. A recent insight about the signaling pathway of flaviviruses, which drives the primary steps of their infection has been successful in providing a schematic diagram of the biology of these viruses. Genome-wide CRISPR-Cas9 screening has identified nine host genes that are involved in flavivirus infectivity. The endoplasmic reticulum (ER) plays an indispensable role in replication, translation, polyprotein processing, virion morphogenesis and consequently, controlling the life cycle of flavivirus.^7^ In this line, most of the suspicious genes were associated with ER. Studies have elucidated the unique dependency of flaviviridae on ER-associated signal peptidase complex 1 (SPCS1) proteins. Disruption of SPCS1 processing pathway reduced the infection level of all flaviviridae members.^8^ Moreover, orthologous functional genomic CRISPR-Cas9 screening revealed various host factors involved in virus entry (AXL), endocytosis (RAB5C, RABGEF) and transmembrane protein processing and maturation (EMC) which are associated with the infection of the DENV and ZIKV.^9^ TLR7/8 agonist R848 strongly restrains ZIKV replication. It is indicated that replication inhibitory effect of R848 is mediated by viperin, an IFN-inducible protein. To confirm this claim, CRISPR-Cas9 genome editing tool was used to knock out (KO) viperin in human MDM cells, as a result, R848 inhibitory effect relieved in KO-cells.^10^ The emergent outbreak of ZIKV and the complexities of its infection highlight the need for a low-cost sequence-specific diagnostic platform that can be used in pandemic regions. Likewise, the inferior performance of the detection method based on antibodies and their limitations just as encountering problems with off-targets and gaining false positive results of sequence-based diagnostics; make these conventional methods to meet CRISPR-Cas9 technology, as an alternative strategy.^11^ Many strain-specific PAM sites in the Zika strain provide the opportunity to discriminate viral lineages by utilizing a newly established freeze-dried platform termed as ‘Nucleic Acid Sequence-Based Amplification (NASBA)-CRISPR’. As part of NASBA reaction 1) the strain-specific PAM sequence, 2) appropriate gRNA, and 3) the double-stranded DNA are produced and subjected to Cas9-mediated split. The presence of a strain-specific PAM leads to the production of truncated RNA product which lacks the sensor H trigger sequence. Contrary to the full-length RNA, the truncated RNA is unable to stimulate the sensor H toehold. Hence, this method could be employed for detecting the strain-specific lineage of the virus without any contamination from other flavivirus types.^12^

### The therapeutic application of CRISPR-Cas9 technology to human viruses

#### Hepatitis B viruses

CRISPR-Cas9 editing tool presents an alternative approach to uproot HBV replication and abolish its latent viral reservoir, i.e. a form of covalently closed circular DNA (cccDNA), in infected cells. Compared to other potential sites, conserved sequences including C, P, S, and X ORFs in HBV genome are more precedent in order to be used as potential targets for designing gRNA. Owing to minor concordance between the conserved sequences of HBV and human genome, the off-target mutations will restrain on host’s genome while alleviating viral infections simultaneously. Designed gRNAs have been shown to reduce the HBV DNA level from 77 to 98% in cultured cells.^13^ Likewise, designed gRNAs targeting HBV cccDNA in HBV-infected HepG2/NTCP cells has resulted in eight-fold reduction in the expression of HBcAg.^14^ Targeting multiple regions of HBV genome by co-transfection of several gRNAs has been reported to increase the effectiveness of the approach.^15^ A number of studies have been designed to take advantage of introducing large deletions via CRISPR-Cas9 system in combination with the efficiency of lentivirus mediated gene transfer to effectively prevent the HBV replication.^16^ Owing to fact that CRISPR-Cas9 technology can affect the off-target sites, design and characterization of fastidious CRISPR-Cas9 system for more precise targeting of invasive elements should be a matter of focus. In this context, a more accurate form of CRISPR-Cas9 technology, Cas9 nickases (Cas9n), has been proposed for targeting conserved sequences in the S and X ORFs of the HBV genome. This strategy was able to disrupt HBV replication in chronically and *de novo* infected hepatoma cell lines as well as episomal cccDNA and chromosomally integrated HBV target sites.^17^ As a proof of concept, it is required to evaluate the antiviral effect of CRISPR-Cas9 system in more pertinent *in vivo* models of HBV infection.

#### Human immunodeficiency virus

Human immunodeficiency virus (HIV-1) is a major global health problem for which no effectual vaccine is available. The latent reservoir of HIV-1 can persist for as long as 60 years in CD4+ T cells. Purging of HIV-1 reservoirs is the effective cure to obviate the expansion of the virus into healthy cells in patients. Two main strategies are currently followed to cure the HIV-1 infection: 1) a functional cure, in which the viral replication is controlled while latent reservoir still remains; e.g. the impairment of the CCR5 receptors, and 2) a sterilizing cure, in which even viral traces are eliminated from the infected cells.^18^ Individuals carrying a 32-bp deletion in their *CCR5* gene (*CCR5*∆*32*) are instinctively resistant to HIV-1 infection. By transplanting *CCR5*∆*32* hematopoietic stem cells, one can easily devise a sterilizing cure strategy. However, tropism shift to CXCR4 can occur to cope with the impairment of CXCR5. Exploiting CRISPR-Cas9 system can overwhelm this hurdle, because this system has the potential to disrupt CXCR4 without affecting the cell propagation.^19^ Introduction of the homozygous CCR5∆32 mutation in induced pluripotent stem cells (iPSCs) using the combination of CRISPR-Cas9 system and a PiggyBac transposon caused a significant resistance to HIV infection. Also, downstream lineage, the monocytes and the macrophages derived from these engineered iPSCs, represented the same resistance. Therefore, these new established cells could be considered as a source for autologous therapy in HIV infection.^20^ To overcome little activity of CRISPR system in CD4^+^ T cells, it is possible to utilize a dual gRNA approach for inducing biallelic deletion in CCR5 gene and consequently, improve the disruption of CD4+ T cells and CD34^+^ HSPCs.^21^ Recently, Cas9 ribonucleoprotein (RNP) complex has been used to target host factors that are involved in HIV infection. As a result, a tropism-dependent resistance to HIV infection is pointed out in CXCR4 or CCR5 disrupted T-cells. Remarkably, simultaneous targeting of CXCR4 and CCR5 by CRISPR-Cas9 system, significantly decreased tropic-dependent HIV-1 in CXCR4- and CCR5-modifed cells (TZM-bl cells, Jurkat T cells, and human CD4^+^ T cells) without any cytotoxic effects on cells viability.^22^ Moreover, targeting the factors that are involved in later stages of initial HIV infection, such as LEDGF or TNPO3, represented a tropism-autonomous diminution in infected T-cells.^23^ Furthermore, CRSPR-based genetic screen discovered that three host dependency factors (*TPST2*, *SLC35B2*, and *ALCAM*) play vital roles in HIV infection in primary CD4^+^T cells.^24^ In order to target the proviral DNA efficiently, it is utterly crucial to eradicate the viral remnant sequences from cells completely. Long terminal repeat (LTR) is an important element in augmenting transcription of potentially toxic proteins in HIV infectivity. To eliminate the entire viral genome, recruiting Cas9 simultaneously to 5’ and 3’ LTR will untwist HIV genome from infected cells.^25^ Recently, it was reported that HIV-1 genome can be eradicated from the host genome in 2D10 CD4^+^ T-cells, where CRISPR-Cas9 system was delivered by lentiviral vector to target LTR U3 regions.^26^ In a further attempt, recombinant Adeno-associated virus 9 delivery of SaCas9, a shorter variant of Cas9 derived from *Staphylococcus aureus*, was adapted to excise segments of integrated HIV-1 by targeting within the 5′-LTR and the gag gene in transgenic mouse and rat. This was the first report that clarified the promising results of CRISPR-cas9 system for *in vivo* studies.^27^

What would happen if we design gRNAs targeting non-conserved regions in HIV-1 genome? The question was answered recently in CD4^+^ T cells expressing Cas9 and gRNA ceaselessly. It is elucidated that targeting non-conserved regions resulted in noticeable obstacle of the infection in transient assays but after a variable time all targeted infections came up with a high level of HIV-1 production. Moreover, after a longer time, targeting conserved regions in HIV-1 genome showed an escape as well. Genome sequencing of escaped viruses has disclosed that the gRNA binding site and PAM region in HIV-1 genome were eradicate by some mutations that were introduced by error-prone NHEJ repair pathway.^28^ Several approaches can be used to vanquish HIV-1 escape including multiplex targeting by designing strong gRNAs to direct Cas9 on conserved regions,^29^ utilizing Cas9 variants that recognize different PAM formats,^30^ using CRISPR-like enzyme such as Cpf1 that introduces cut in the distal site of the binding site,^31^ and abrogation of NHEJ by chemical drugs for instance SCR7.^32^Table 1 shows CRISPR-Cas9 targeting sites in other virus infections.

#### Delivery of CRISPR-Cas9 components

Despite investing considerable effort in gene therapy during the last decades, limited success has been achieved due to the shortcomings of existing viral and non-viral gene delivery approaches. Generally, viral delivery systems can be categorized into four main classes 1) adenoviruses, 2) adeno-associated viruses (AAV), 3) retroviruses, and 4) lentiviruses. Lentiviruses are derived from HIV-1 and have the potential to cause undesirable modifications in long-term expression cell lines. Thus, integrase-defective lentiviruses which are replication incompatible or at least single-cycle replicable are more preferred. This preference is more prominent in the case of genome editing that requires long-term expression of the genome editing components and engages an increased risk of unwanted off-target changes.^33^ CRISPR-Cas9 system packaged with lentiviral vectors has shown promising results in eliminating latent HIV-1 infection. Moreover, prepackaged Cas9 in a transient form of virus-like particles that target CCR5 represents a reduced off-target effect in target cells.^34^ Recombinant AAV vectors have low pathogenicity and low immunogenicity compared to other viral vectors, but their main obstacle is their limited packaging size. The size limitation of AAV vectors can be overcome by exploiting *SaCas9* (3.3 kb) or by using split-Cas9 approaches.^35^*In vivo* genome editing generally requires an effective method to deliver the components of editing tools appropriately. For the first time, it was demonstrated that delivering multiplex saCas9/sgRNA, targeting two LTR sites and two structural proteins, in an all-in-one AAV–DJ/8 vector can be applied to precisely excise HIV-1 proviral from pre-clinical mouse models.^36^ This strategy is a promising approach to eradicate even trace of proviral in different organs by simultaneously introducing indels and large deletions at HIV-1 reservoirs. Despite the high productivity of viral vectors, certain limitations such as immunogenicity and random integration of conventional viral vectors led the studies to fluctuate delivery approaches with a view to non-viral gene delivery.^37^ So far, different classes of non-viral vectors have been introduced. Non-viral expression plasmid is the most convenient delivery approach that can express CRISPR-Cas system in a safe mode. However, random integration of the plasmids and difficulty in controlling their timeframe expression are the main obstacles. To address these drawbacks, CRISPR mRNA delivery system has been employed, which illustrates great refinement in decreasing the risk of off-target activity by controlling the amount of Cas9 protein and gRNA level.^38^ Besides, rapid deterioration of plasmid and mRNA by serum nucleases is another major hurdle that must be resolved. The use of RNPs is another approach to delivering Cas9-gRNA with higher control on editing timeframe. Delivering RNPs by using electroporation method has showed promising results in targeting host factors that are involved in HIV-1 infected CD4^+^ T cells.^23^ However, some complications such as the negative charge of RNAs, flimsy structure, and the large molecular size of proteins limit the diffusion rate of RNPs across the cell membrane. To overcome reduced delivery efficiency of non-viral delivery platforms, positively-charged nano-carriers can be employed as an ideal delivery system. Yarn-like DNA nano-clew is a form of cationic nano-carriers which can be loaded with CRISPR-Cas9 technology to shuttle Cas9-gRNA into the target cell. This method provides stability between binding and discharge of the CRISPR-Cas9 system.^39^ Microfluidic membrane deformation (MMD) through the transient disruption of the cell membrane has been exploited as a Cas9-gRNA delivery platform. Similar to microinjection, MMD can deliver payload across different cell types even hard-to-transfect cells, but in an easier manner with a higher yield. Moreover, MMD has portrayed more cell viability than electroporation method. Collectively, MMD seems to warrant a precise and efficient genome editingapproach.^40^

**Table 1:** CRISPR-Cas9 targeting sites in different virus infections. Applying CRISPR-Cas9 technology to target virus genomes and to find signaling pathways that are involved in virus infections

**Virus**	**Gene target site in virus/Human**	**Model**	**Delivery**	**Reference**
**EBV**	EBNA1, LMP1, EBNA3C	Burkitt’s lymphoma cell lines Raji cell	Transfection	^41^
	BVRF1	Gastric Cancer Cell line, SUN719 and YCCEL1	Transfection	^42^
	BART5, BART6, or BART16	gastric carcinoma cell line SNU-719	Transduction	^43^
**HTLV1**	pX region	ED T-Cell	Transduction	^44^
	RNF8	HeLa cells	Electroporation	^45^
**JCV**	T-antigen	Human oligodendroglioma cell line, primary human fetal glial cells	Transfection	^46^
	NCCR-a and VP1-b	glial derived SVG-A cells and human fetal kidney derived hTERT transformed HuK(i)G10 cells	Transduction	^47^
**HPV**	E6	SiHa and CaSki cells (cervical carcinoma cell lines)	Transfection	^48^
	E7	SiHa and Caski cells	Transfection	^49^
	E7	HeLa cells	Nano-micelle	^50^
**HSV**	UL8, UL29, and UL52	Vero cells	Transduction	^43^
	ICP0	HEK293T cells	Transduction	^51^
	UL7	HEK293T cells, Vero cells and BALB/c mice	Transfection	^52^
**HCV**	5′-UTR and 3′-UTR regions	Huh-7.5 cells	Transfection	^6^
	ISG15	U2OS cells	Transfection	^53^
	miR-122	Huh-7.5 cells	Transfection	^54^
	miR-122/hcr locus	hepatoma cells	Transfection	^55^

EBV: Epstein–Barr virus, HTLV1: Human T-lymphotropic virus 1, JCV: JC virus, HPV: Human papilloma virus, HSV: Herpes simplex virus, HCV: Hepatitis C virus, EBNA1: Epstein–Barr Nuclear Antigen 1, LMP1: Latent Membrane Protein 1, EBNA3C: Epstein–Barr nuclear antigen 3C, BVRF1: DNA packaging tegument protein UL25 of EBV, BART5, BART6, or BART16: BamHI-A rightward transcript 5, 6 or 16, pX region: A region of HTLV1 genome which encodes regulatory and accessory genes, RNF8: Ring Finger Protein 8, T-antigen: Large tumor Antigen, NCCR-a: non-coding control region-a, VP1-b: Viral Protein 1, E6 and E7: Early proteins 6 and 7, UL8, UL29, UL52 and UL7: Unique Long 8, 29, 52 and 7, ICP0: Infected-Cell Protein 0, 5′-UTR and 3′-UTR: 5′ and 3′ Untranslated Region, ISG15: Interferon Stimulated Genes 15, miR-122 : microRNA-122, hcr locus: hepatocellular carcinoma related locus

## Conclusion

The versatility and feasibility of the CRISPR-Cas9 system remove some of the impediments that has challenged gene therapy approaches and introduce new opportunities in antiviral therapies. Despite the massive growth spurt of CRISPR-Cas9 technology over the last years, major efforts are needed to address the remaining impediments and develop CRISPR-Cas9 based safe delivery technologies. Further studies are required to investigate the immune responses to exogenously expressed CRISPR-Cas9 system and devise strategies to mask this system and thus reduce their immunogenicity. High-fidelity Cas9 variants introduced their efficacy in the field of genome editing by reducing off-target effects.^1^ Application of these variants to eradicate viral infection from host genome may bring forth new perspective. Viral and non-viral delivery systems have their own drawbacks when applied in gene therapy approaches. Recent studies have shown that by combining lipid nanoparticle-mediated delivery of Cas9 mRNA and AAVs encoding gRNA and donor template, efficient *in vivo* restoration of > 6% can be achieved in a mouse model of human hereditary tyrosinemia.^37^ The combination of these two conventional delivery methods could pave the way for curing viral infections in clinical settings.

## Acknowledgments

Authors wish to thank School of Advanced Technologies in Medicine, Shahid Beheshti University of Medical Sciences for its support.

## Ethical Issues

Not applicable.

## Conflict of Interest

The authors declare no conflict of interests.
